# Preliminary efficacy of a transdiagnostic parent-led internet-delivered intervention for children with anxiety and depressive symptoms: a pilot randomized controlled trial

**DOI:** 10.1186/s13034-024-00721-3

**Published:** 2024-03-06

**Authors:** Costina-Ruxandra Poetar, Anca Dobrean, Gerhard Andersson

**Affiliations:** 1 Department of Clinical Psychology and Psychotherapy, Babeș-Bolyai University, Cluj-Napoca, Romania; 2 The International Institute for the Advanced Studies of Psychotherapy and Applied Mental Health, Babeș-Bolyai University, Cluj-Napoca, Romania; 3 Department of Behavioural Sciences and Learning, Linköping University, Linköping, Sweden; 4 Department of Biomedical and Clinical Sciences, Linköping University, Linköping, Sweden; 5 Department of Clinical Neuroscience, Karolinska Institute, Stockholm, Sweden

**Keywords:** Transdiagnostic, Internet interventions, Anxiety, Depression, Children, Parents

## Abstract

**Background:**

There is extensive research indicating that transdiagnostic interventions are effective for adults and children with anxiety and depressive disorders; however, limited research has been conducted on the efficacy of such programs targeting parents of children with anxiety and depressive symptoms delivered via the Internet. This study aims to investigate the preliminary efficacy of a transdiagnostic Internet-delivered intervention for Romanian parents of children with elevated anxiety and depressive symptoms.

**Methods:**

We conducted a single-blinded pilot randomized controlled trial. Forty-two parents of children with anxiety and depressive symptoms (*M*_*age*_ = 39.79, 78.6% women) from Romania were randomly assigned to one of the two groups, to ParentKIT, an Internet-delivered intervention based on Rational Emotive and Behavioral Therapy (*n* = 21), or to a delayed treatment condition (waitlist group) (*n* = 21). ParentKIT consisted of a brief transdiagnostic intervention delivered through nine modules with therapist guidance.

**Results:**

A significant Group by Time effect was found for child emotional problems as reported by parents (Cohen’s *d* = − 0.85) and for parental self-efficacy (Cohen’s *d* = 0.71).

**Conclusion:**

A transdiagnostic Internet-delivered intervention addressed to parents of children with elevated anxiety and depressive symptoms is a promising approach. Future research should include blind assessments as well as multiple outcome assessors and investigate the long-term efficacy of the intervention.

**Trial registration:**

NCT05341297.

## Introduction

Anxiety disorders are the most common mental health problems in children and adolescents (from now on children) worldwide [1], with a prevalence of 6.5% of any anxiety disorder. As most mental disorders arise in childhood, 51.8% of anxiety-related disorders emerge before the age of 18 years [2]. Often anxiety disorders are comorbid with depressive disorders [3], which means more severe symptomatology, more chronicity, and the need to tailor the treatment. Psychological and pharmacological treatment is available for children with anxiety and depressive disorders, with long-standing evidence that Cognitive Behavioral Therapy (CBT) is an effective intervention, as indicated by several meta-analyses [4–6]. However, only a small percentage of children faced with these mental health problems access treatment. Several explanations for this gap in access to treatment could be related to attitudes, knowledge about mental health problems and available treatments, financial barriers, or lack of specialists [7, 8].

Frequently anxiety and depressive disorders go undetected in children [9], and even when diagnosed a small percentage get access to treatment (e.g., 30% in the UK). In fact, up to 90% of young people in some developing countries do not have access to treatment [10, 11]. Access to mental health services is even scarce in developing countries and, for instance, in the case of some mental health problems (e.g., Attention-Deficit/Hyperactivity Disorder), pharmacological treatment may be more available to parents, than psychotherapy [12]. Internet-delivered interventions could overcome existing gaps in access to treatment, being effective for both adults with anxiety and depressive symptoms [13], and adolescents with anxiety symptoms [14], however, access to such programs in developing countries is limited, with most of the Internet-delivered programs developed in high-income countries (e.g., Australia, USA, Sweden, UK [14]).

Another important aspect to consider is the fact that a significant percentage of children (e.g., up to 60% of depressed youth) do not respond to such interventions [15, 16], and a possible explanation could be that these interventions are diagnostic-specific, targeting one condition at a time, while not focusing on comorbid problems. Given the fact that the comorbidity is high between anxiety and depressive disorders [3], and that certain symptoms can trigger others, as indicated by a recent study investigating the network connection between anxiety and depressive symptoms [17], there is a pressing need for transdiagnostic interventions that address multiple conditions simultaneously. Transdiagnostic approaches for youth are effective [18–20] in reducing child anxiety and depression; however, there are very few interventions available through the Internet, most of which are limited to feasibility studies or open trials [21, 22]. Moreover, existing transdiagnostic Internet-delivered interventions primarily target adolescents, with only one study, to the best of our knowledge, focusing specifically on younger children [23]. This highlights a critical gap in accessible and age-appropriate mental health support for this vulnerable age group through Internet-delivered interventions.

Parents could be involved in Internet-delivered interventions when they recognize that their children are struggling with anxiety or depressive disorders. They can provide support and access to mental health services. It is not clear whether parental participation in remote CBT interventions for youth improves outcomes [24], however, this may be due to the high heterogeneity in what parental involvement means in such interventions. On the contrary, parent-led interventions are an alternative to child-focused CBT interventions, focusing on empowering parents to manage child anxiety by reducing specific risk factors involved in the maintenance of avoidance behavior (e.g., family accommodations) [25]. Furthermore, parent-led interventions could surpass stigma gaps associated with receiving mental health treatment and serve as an acceptable treatment option when children refuse treatment [26]. Parent-led interventions can be effective in treating child anxiety [27, 28], with results from a meta-analysis showing a small effect size compared to waitlist control [29]. Data from a randomized controlled trial indicated that parent-led CBT is as effective as therapist-delivered CBT for child anxiety [25]. In this study, the parent-led intervention was noninferior to CBT in the outcomes related to child anxiety, however, the reductions in parental accommodations were higher in the parent-led group.

While this existing research on parent-led CBT indicates it is a promising approach, including maintained or even improved treatment gains at follow-up assessments [29], an important endeavor is to expand research to evaluate the efficacy of parent-led interventions for child depression outcomes as well and for alternative delivery methods (e.g. Internet-delivered treatment). Recently, online delivery methods for parent-led CBT have been investigated [30]. In this study, were involved parents of children aged 8–9 years who screened positive for anxiety disorders in a school assessment. The intervention consisted of 8 modules (20–30 min), telephone support (up to 20 min) for each of these modules for parents, as well as a mobile game application for children (Monster’s Journey: Facing Fears) [30].

Parent-led CBT has shown promise in treating child anxiety, but there is limited research investigating the efficacy of scalable solutions to deliver such interventions. Incorporating transdiagnostic approaches into these interventions could lead to significant reductions in depressive symptoms. So far, there is a scarcity of studies that evaluate parent-led transdiagnostic interventions through technology. A single study [28] was identified that investigated the efficacy of a 6-week parent-led transdiagnostic CBT intervention for children with emotional problems related to the COVID-19 pandemic. The intervention delivered by videoconferencing showed significant reductions in parental ratings of child anxiety, depression, stress, and improvements in family relationships. Given the promising results of this study in terms of outcomes, response rates (62%), lower dropout rates (21%), and increased parental satisfaction with the intervention, as well as considering the limitations of this study, such as the lack of a control group, we consider that much research is needed to investigate the efficacy of delivering parent-led CBT interventions.

Furthermore, recent evidence from a study conducted with adults indicated the relationship between the accommodation of symptoms and the severity of depression [31]. Therefore, parent-led CBT focusing on accommodations could be a promising approach to treating children with anxiety and depressive symptoms. Furthermore, transdiagnostic parent-led interventions delivered via the Internet could enhance access to evidence-based mental health interventions. Even though previous studies involved only parents of younger children, adolescents face the worst access to mental health services [32]. Consequently, parent-led CBT could be particularly suitable as a low-intensity intervention for this population as well.

Recent evidence suggests that briefer interventions can be effective. For instance, a single-day parent-only intervention has been shown to produce comparable results in reducing anxiety in children and their siblings, as reported by parents, when compared to a 6-week group intervention [33]. However, no investigations have been conducted on the efficacy of a brief parent-led transdiagnostic Internet-delivered intervention.

The primary objective of the present study was to examine the preliminary efficacy of a parent-led transdiagnostic Internet-delivered intervention developed for Romanian parents of children with anxiety and depressive symptoms. We hypothesized that participants in the ParentKIT group would report lower scores on child emotional problems at posttest as compared to the control group and higher scores on parental self-efficacy regarding parenting practices associated with reduced risks of child anxiety and depressive symptoms. Exploratory, we aimed to investigate the preliminary efficacy of the program in reducing parental distress. Furthermore, we hypothesized that the parents’ satisfaction with the intervention was high.

## Methods

### Participants

Participants were recruited between March 2022 and June 2022 through online advertisements, such as paid social media campaigns promoted in Facebook groups dedicated to parents, as well as through the project’s website, where we had provided information about the study and an easy online sign-up option for interested participants.

Inclusion criteria: (1) be the parent of a child/ adolescent aged between 6 and 14 years old, (2) be able to read and write in Romanian, (3) report elevated child symptoms of anxiety and/or depression symptoms, and (4) have access to the Internet.

Exclusion criteria: child currently undergoing concurrent treatment (psychotherapy sessions, pharmacological treatment).

The required number of participants was calculated a priori using G*Power [34] which indicated that the minimum required sample size was 42 participants based on a medium effect (repeated measures between factors, *f* = 0.50, *α* = 0.05, power 0.95, correlation between repeated measures = 0.50).

**Fig. 1:**
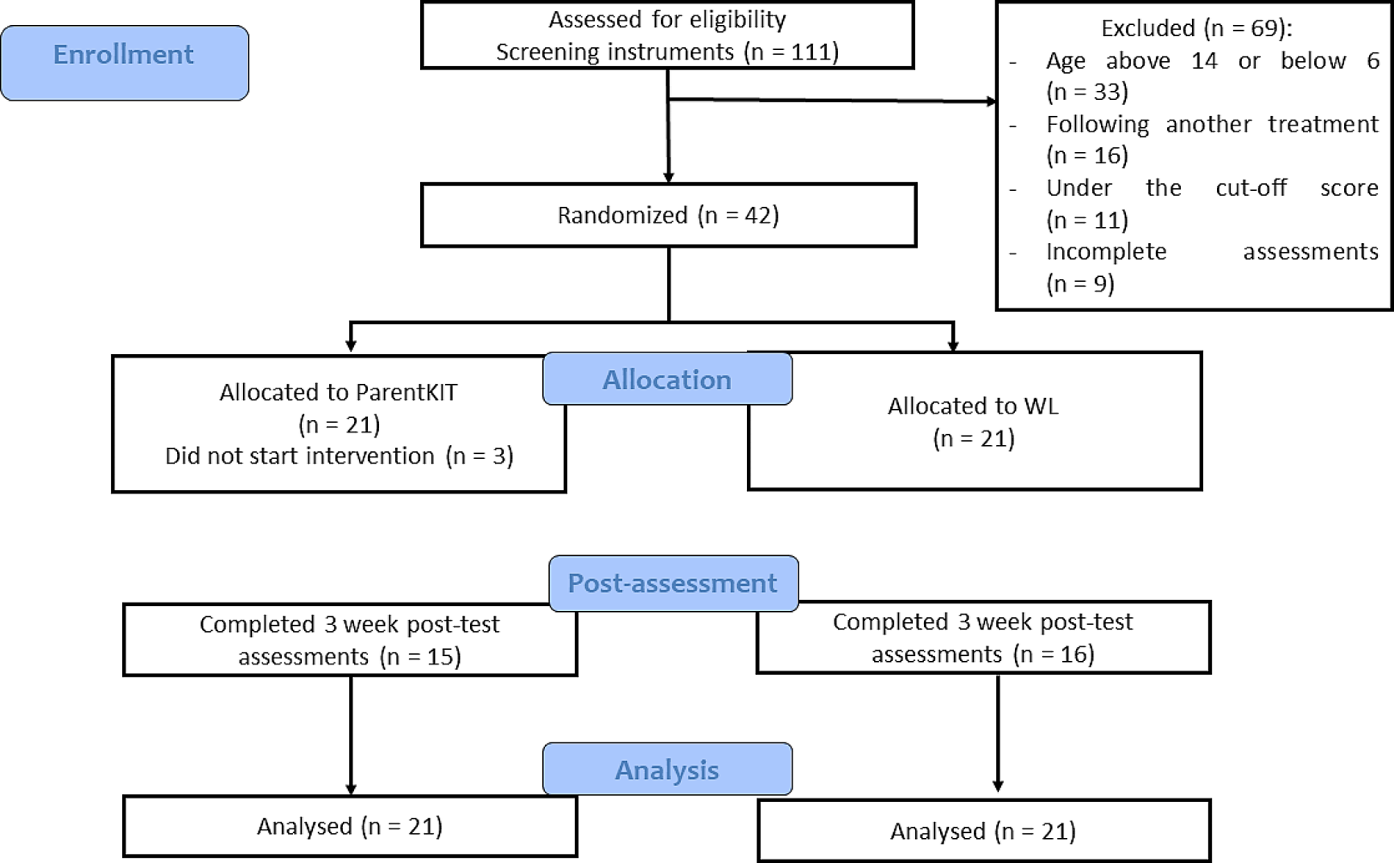
CONSORT diagram of the pilot randomized controlled trial

A total of one hundred eleven parents expressed interest in taking part in the study. Forty-two parents (*M*_*age*_ = 39.79, *SD* = 2.81, 78.6% mothers, *M*_*Child age*_ = 10.33, *SD* = 2.59), whose children exhibited anxiety and depressive symptoms, met the eligibility criteria for the study. Figure 1 depicts the CONSORT diagram outlining the flow of participants throughout the study.

### Procedure

The study received approval from the Ethics Committee of the Babeș-Bolyai University. The recruitment of participants took place through online channels, with parents consenting to participate by signing an informed consent form and undergoing a preliminary screening assessment. Eligible participants were randomly assigned in a 1:1 ratio using a random number generator (random.org) in one of the two groups: ParentKIT (*n* = 21) and the waitlist control group (*n* = 21), using a computer generated allocation sequence. The randomization procedure was performed by an independent researcher not involved in the study.

Participants assigned to the experimental group received additional support, as a research assistant contacted them by phone to assist in establishing an account on a secure Iterapi platform [35]. After a three-week intervention period, participants were invited to partake in post-intervention evaluations. Participants were not compensated for their participation.

### Measures

#### Demographic information

A demographic questionnaire was used to collect information about participants, including age, gender, occupational status, marital status, as well as the age and gender of their child.

#### Primary outcome

##### Child’s emotional problems

The parent version of the Strengths and Difficulties Questionnaire for ages 4–17 (SDQ) [36] was used to measure children’s anxiety and depressive symptoms, as reported by parents. It consists of 25 items rated on a three-point Likert scale (0 - *Never true*, 2 - *Very true or very often true*). Sample items are: “Often unhappy, depressed or tearful”, “Many fears, easily scared”. Scores can be computed on a scale of total mental health problems or on five subscales (Emotional problems, Conduct problems, Hyperactivity, Peer problems, and Prosocial behaviors). In the current study, we used the emotional problems subscale. The scores for this subscale range from 0 to 10, and higher scores indicate greater emotional problems. Cronbach’s Alpha in the present study was 0.87 for the Emotional problems scale. The psychometric properties of this scale are satisfactory as indicated in a review in which it showed convergent validity and adequate internal consistency for the total scale [37]; it was also previously used with Romanian parents [38].

#### Secondary outcomes

##### Parental distress

We utilized the Patient Health Questionnaire-4 [39] to assess parental anxiety and depressive symptoms. This scale comprises four items, each rated on a 5-point Likert scale ranging from 0 (*Never*) to 3 (*Always*). Parents must rate their answers to the next question: “Over the last two weeks, how often have you been bothered by the following problems?”, sample items: “Feeling nervous, anxious or on edge”, “Feeling down, depressed, or hopeless”. Scores can range from 0 to 12, and higher scores indicate greater distress. Previous studies reported excellent psychometric properties. This is supported by a systematic review that analyzes the psychometric properties of PHQ-4, which showed adequate reliability for distress and both anxiety and depression, acceptable stability over time, and demonstrated convergent validity [40]. Cronbach’s alpha in the present study was 0.92.

#### Parental self-efficacy

The Parental Self-Efficacy Scale (PSES) [41] was employed to evaluate parental self-efficacy concerning parenting practices aimed at reducing anxiety and depression risks among children and adolescents. The scale was obtained from the original author, following which it was translated and adapted into Romanian by the primary author for the purpose of this study. It comprises nine items, each rated on a 4-point Likert scale (1 = *Not at all confident*, 4 = *Very confident*). Sample items: “How confident do you feel about your ability to find a balance between being involved in your teenager’s life and encouraging age-appropriate independence?”, “If you noticed a persistent change in your teenager’s mood or behavior, how confident do you feel about your ability to help your teenager seek appropriate professional help?”. Good internal consistency has been reported (McDonald’s omega = 0.91), as well as convergent and concurrent validity [41]. Scores can range between 9 and 36, higher scores indicate a higher self-efficacy. Cronbach’s alpha in the present study was 0.96.

#### Satisfaction with the program

We assessed satisfaction with the program by administering the Client Satisfaction Scale (CSQ-8) [42]. Responses are rated on a four-point Likert scale, ranging from 1 *(Not at all satisfied)* to 9 (*Very satisfied)*. Sample items: “How would you rate the quality of the treatment you have received?”, “If a friend were in need of similar help, would you recommend this treatment?”. The answers are rated on a four-point Likert scale. Total scores can range from 8 to 32, higher scores indicating higher treatment satisfaction. This scale showed adequate reliability in other clinical trials investigating the efficacy of Internet-delivered CBT [43]. Cronbach’s alpha in the present study was 0.90.

### Intervention

ParentKIT, an Internet-delivered Intervention, was created based on the fundamental principles of Rational Emotive Behavior Therapy (REBT) [44]. REBT is one of the important tenets of CBT, considered one of the earliest approaches within this treatment orientation [45]. It is considered a transdiagnostic approach [46] and its core mechanisms, namely irrational beliefs, are positively related to both internalizing (e.g., anxiety, depression) and externalizing (e.g., anger) problems, as indicated by the results of a meta-analysis [47].

**Fig. 2:**
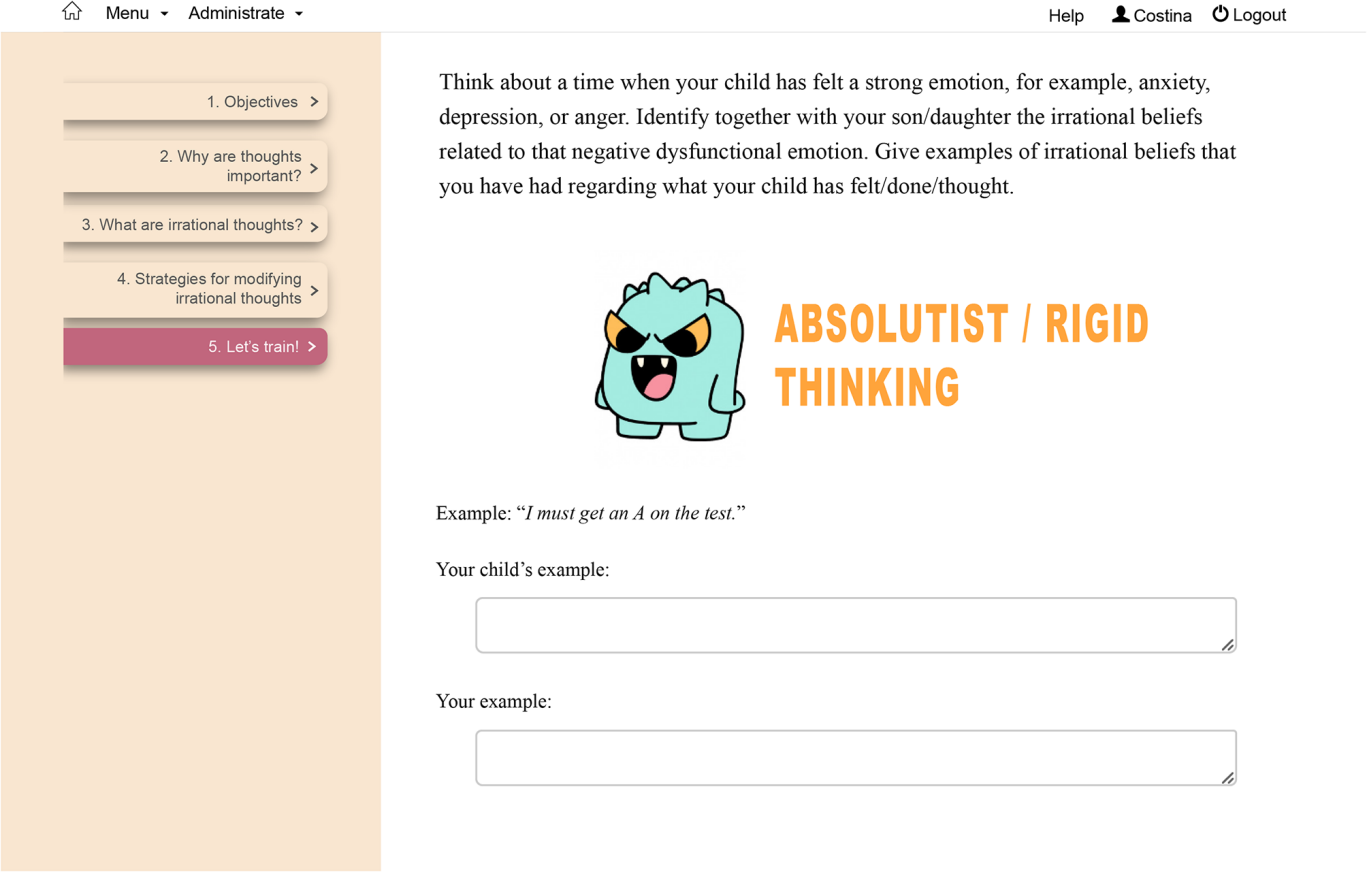
Screenshot of an exercise from Module 7 from ParentKIT

ParentKIT consists of nine modules delivered over three weeks (see Table 1 for the key aspects covered in each module). The content of the nine modules was developed based on REBT theory and treatment techniques. In REBT, a main concept is irrational beliefs, which are involved in dysfunctional emotions and maladaptive behaviors as proximal causes. Through REBT, parents learn to identify irrational beliefs such as demandingness (“I must comfort my child when he is anxious”), catastrophic thinking (“It’s terrible that my child is anxious”), low frustration tolerance (“I cannot stand that he is so worried all the time”) and global evaluation of human worth (“I’m a bad parent”). In the program, parents learn to support their child with anxiety and depression, not participate in family accommodations, and cope with the child’s distress responses by practicing a rational thinking style. Parents discover ways to assist their children by helping them identify thinking errors (irrational thinking) (see Fig. 2 for a screenshot of the platform, which shows an example exercise designed for parents and children to collaboratively identify irrational beliefs), disputing these cognitions and changing them with their rational counterparts such as preferential, flexible thinking (“I would like my child not to be so anxious, but this does not mean it must happen exactly as I want”), non-catastrophic thinking (“It’s not the worst thing that could happen to me”), frustration tolerance (“I dislike this aspect”), unconditional acceptance of self, others and life (“I am a valuable human being”) instead of rigid thinking.

**Table 1:** Module contents

Module name	Strategies	Module content
Module 1. Introduction	Psychoeducation regarding the program	Introduction to ParentKIT Review information regarding the program, therapeutic orientation Identify program goals
Module 2. Anxiety disorders	Psychoeducation regarding anxiety disorders	Present the difference between fear and anxiety Present symptoms of different anxiety disorders Present causes of anxiety disorders Discuss the role of parental factors in anxiety disorders
Module 3. Depressive disorders	Psychoeducation regarding depressive disorders	Present the difference between sadness and depression, how depressive disorders can be recognized Present causes of depressive disorders Discuss the role of parental factors in depressive disorders
Module 4. Treatment options	Psychoeducation regarding existent treatment options	Learn about evidence based treatments Present NICE guidelines recommendations for anxiety and depressive disorders treatment in children
Module 5. Relaxation	Breathing and relaxation exercises	Present the role of relaxation exercises Learn breathing and relaxation exercises Create a relaxation plan to implement with the child
Module 6. Adaptive behaviors	Behavioral activation and exposure	Create an exposure hierarchy Present the relationship between involvement in activities and depressive symptoms
Module 7. Thinking patterns	Identifying, disputing and changing irrational beliefs	Learn the relationship between irrational beliefs and distress Identify irrational beliefs Explore techniques for challenging irrational thoughts and fostering the practice of rational thinking in children
Module 8. Problem solving	Practice problem solving	Discuss the distinction between emotional and practical problems Learn a problem solving approach
Module 9. Prepare for future	Maintaining the progress achieved through the Internet-delivered intervention	Reflect on the strategies presented during this program Set long-term objectives to sustain progress Plan for future stressful situations

In addition to the cognitive restructuring module, parents are provided with strategies to manage anxiety and depressive symptoms (e.g., exposure, behavioral activation, problem solving, relaxation) and techniques to manage their own psychological distress. Parents were instructed to provide assistance and autonomy, create a supportive environment, recognize and validate their child’s feelings, be consistent in providing feedback, identify and prevent obstacles in applying techniques, remove accommodations related to anxiety and depressive symptoms.

Each module adhered to a well-defined structure consisting of various specific sections in each session: module objectives, the central focus of this module, summary, and homework assignment. To enhance the user experience, each module incorporated text and video resources. The video materials included either experts explaining the concepts, rationale for applying a certain technique, and strategies involved in the treatment process or depicting parent-child interactions as they applied the techniques. Furthermore, participants had access to brochures that they could download and print, providing them with additional information and support. Finally, parents could download a diploma for participating in this program.

ParentKIT was facilitated by two psychotherapists undergoing supervision training in Cognitive Behavioral Therapy, who were trained by the first author on the treatment protocol. Therapists provided valuable feedback on the modules and homework worksheets, while also providing support and addressing any queries parents had. The intervention program was designed to be flexible. Parents were given the autonomy to choose when to engage with the content, and each set of modules remained accessible for a period of seven days, allowing parents time to engage with the content before new modules became accessible. During the seven days, parents could read the assigned modules, practice the skills, and complete homework. They could also address their allocated therapist questions regarding clarification using the chat available on the platform. Access to previous modules was not removed, and parents could review previous modules at any time.

#### Control group

Participants allocated to the control group had access to the online platform where they completed the assessments. This was a delayed treatment group (waitlist control, WL), with participants accessing the treatment modules after three weeks. Participants were informed that they were in this group and would have access to the intervention after three weeks.

### Statistical analysis

Data were analyzed using RStudio [48]. Hypotheses were tested using a linear mixed model, while missing data was dealt with using multiple imputation by chained equations (MICE), which is the state of the art method to manage missing values [49]. MICE uses chained equations to generate plausible values for missing data based on available data [49]. To incorporate in the estimates the inherent uncertainty of the missing values, the estimates are aggregated across multiple sets of complete data [49]. In our case, the estimates were aggregated across 100 sets of imputed data using Rubin’s rule [50]. The confidence intervals for each estimate were computed based on the aggregated estimate values and their aggregated standard error using the formula: estimate +/- standard error of the estimate multiplied by 1.96 (z-value corresponding to *p* = .05, two tailed). Mixed models analysis with multiple imputation was performed in RStudio using the R-package “mitml” [51].

For effect size data, we used Cohen’s *d* [52] where effect sizes are classified into three categories, namely small (*d* = 0.20), moderate (*d* = 0.50), large effect size (*d* = 0.80).

## Results

### Participants

The demographic characteristics of the included participants are presented in Table 2.

**Table 2:** Demographic characteristics of the participants at baseline

	ParentKIT (*n* = 21)	Waitlist (*n* = 21)
Characteristics		
**Demographic data**		
Parent age (years) (SD)	40.43 (3.01)	39.14 (2.61)
Parent Gender (% female)	17 (81%)	16 (76.20%)
Child age (SD)	10.86 (2.43)	9.81 (2.69%)
Child Gender (% female)	14 (66.7%)	12 (57.1%)
**Parental marital status**		
Married	21	20
Divorced	0	1
**Parent’s highest education**		
Highschool	2	4
Bachelor’s degree	9	9
Master’s degree	10	8
**Parent’s employment**		
Full-time	19	20
Part-time	1	1
Unemployed	1	0
**Residency**		
Urban	18	18
Rural	3	3
**Previous diagnosis**	4	3
Primary diagnosis anxiety disorder	3	2
Primary diagnosis depressive disorder	1	1
**Previous treatment**		
Psychological treatment	4	3

Note. M = mean; SD = Standard Deviation; WL = Waitlist

### Adherence

A high proportion of parents completed post-intervention assessments (73.80%). On average, participants completed approximately six of the nine modules available (*SD* = 3.06). Participants’ engagement with the modules varied, spanning from a minimum of one to a maximum of nine modules read. Twelve participants (66.66%) completed more than four modules, while only seven participants completed the nine modules (38.9%).

### Preliminary efficacy

#### Primary outcome

Child anxiety and depressive symptoms as reported by parents.

Regarding emotional problems of the child as reported by parents, the results showed a significant effect for Group by Time, *b* = -1.90, 95% CI [-3.00, -0.77], *t* = -3.34, *p* = .001, indicating that the participants in the ParentKIT group improved more than the participants of the control group. The between-group treatment effect was Cohen’s *d* = -0.85, 95% CI [-1.46, -0.25], indicating a large effect size. The means and standard deviations for the outcomes at both assessment points (baseline and postintervention) are presented in Table 3.

**Table 3:** Estimated Means (Standard Deviations) for primary and secondary outcomes at pretreatment and post-treatment on parent reports

	T0: Baseline		T1: Post-treatment	
	ParentKIT	Waitlist	ParentKIT	Waitlist
Emotional problems	7.00 (1.76)	6.48 (1.40)	5.32 (1.83)	6.83 (1.80)
Parental self-efficacy	24.67 (3.12)	24.57 (4.26)	26.95 (3.79)	23.60 (6.36)
Parental distress	4.57 (1.91)	4.52 (2.37)	3.92 (3.66)	3.71 (2.56)

#### Secondary outcomes

##### Parental self-efficacy

In terms of parental self-efficacy in reducing the risks associated with child anxiety and depression, the results indicated a significant Group by Time interaction, *b* = 3.07, 95% CI [0.60, 5.33], *t* = 2.57, *p* = .010. The between-group treatment effect was Cohen’s *d* = 0.71, 95% CI [0.09, 1.34], indicating a large effect size in favor of the ParentKIT group.

##### Parental distress

No significant interaction effect was found for parental distress, *b* = 0.16, 95% CI [-1.29, 1.76], *t* = 0.21, *p* = .831.

##### Satisfaction with the intervention

Participants expressed a high level of satisfaction with the intervention, *M* = 26.81, *SD* = 3.01.

## Discussion

The aim of the present pilot study was to present the preliminary efficacy of a newly developed transdiagnostic parent-led Internet-delivered intervention for parents of children with anxiety and depressive symptoms. Previous research indicates that parent-led CBT is a promising approach to treating children with anxiety disorders [29]. However, given the high comorbidity between anxiety and depressive disorders [3], and the promising effects of transdiagnostic Internet-delivered interventions [13], our objective was to develop and test a digital tool in the treatment of internalizing problems.

Our results indicate that parent-reported child internalizing problems were reduced in the ParentKIT group and that satisfaction with the intervention was high. These findings replicate other research findings on the efficacy of parent-led CBT interventions delivered face-to-face or telepsychiatry format, which showed significant decreases in child anxiety as reported by parents and a high level of satisfaction with the intervention [27, 28]. While a previous transdiagnostic parent-led study [28] separately reported anxiety and depressive outcomes given the fact that they used different psychological instruments, we could not directly compare the effect size obtained on our primary outcomes as we used an emotional problems subscale. However, while in the Guzick study [28] the pre-post effect-sizes were in the moderate range for anxiety (*d* = 0.56) and depressive outcomes (*d* = 0.69) as reported by parents, the effect sizes for emotional problems in our study were in the moderate to large range. However, dropout rates were slightly higher in our study, as compared to other studies where only 21% participants dropped out [28]. This aspect is of particular importance to address in future studies, as while we delivered a briefer intervention consisting of 3 weeks, other parent-led CBT interventions delivered such interventions for 6 weeks [28], 8 weeks [27], or 12 weeks [25]. Additionally, our approach differed by not incorporating synchronous interaction between parents and therapists, a contrast to other studies that relied on direct contact via videoconferencing [28] or face-to-face meetings, including some type of telephonic communications [25, 27]. Given that our intervention did not include telephone or face-to-face contact, it is possible that parents’ efforts were lower, as indicated by other studies conducted with other populations (e.g., parents of children with ADHD [53, 54]).

Parents’ self-efficacy in managing anxiety and depressive symptoms in their children also increased from baseline to posttreatment. This is similar to previous research [55], indicating that parental self-efficacy can be a modifiable factor that can be targeted by Internet-delivered interventions. Given that our intervention was brief (9 modules delivered over 3 weeks), it is possible that parents did not have enough time to complete all modules before post-treatment assessments. To ensure adequate time for practice and completion of all essential components, it might be necessary to extend the duration of the treatment.

Furthermore, since all contacts between parents and clinicians were online, it is possible that some parents need more support.

We did not find changes in parental distress, and this can be explained by the fact that only a small percentage of the included parents qualified for moderate (*n* = 7) to severe distress (*n* = 2), based on the cut-off point of the measure used to assess this construct. Another potential explanation may be related to the fact that the program did not address parental distress directly. Specifically, we did not include a separate module for parental distress. Parents learned about anxiety and depressive disorders in the first two modules and about their behaviors involved in the maintenance of their child’s symptoms. Information about behavioral activation, exposure, and thinking patterns was presented only during the last week of treatment. Therefore, in the case of distressed parents, it is possible that this information would have been more useful if it had been presented earlier. In particular, this could be motivated because high parental distress can be a significant barrier in progress throughout the program.

### Limitations

The results of the present study must be interpreted in light of several limitations. The results of the study may not be generalizable to a larger population of parents of children with anxiety and depression, as the sample size was small and possibly not representative of the population. The study was a pilot RCT focused on immediate outcomes, and we lack information about the long-term effects. Another limitation is related to the assessment instruments used. Clinician-rated outcomes (e.g., clinical rated improvement, diagnosis), multiple reports (e.g., child/ adolescent, teacher) would have been useful. As we relied only on the results reported by parents, child ratings of their anxiety and depressive symptoms would have been useful to investigate the discrepancies between parental ratings and self-rated symptoms. Furthermore, given that we recruited participants online, it is possible that the study suffers from self-selection bias, as participants may have volunteered to participate because they were more interested or motivated to use a digital mental health intervention.

Future research would benefit from replicating our findings using a multi-informant approach. This would involve collecting data from various perspectives to assess the efficacy of this intervention. Specifically, clinician ratings of symptoms and impairment should be considered at baseline, post-intervention, and at various follow-ups. Additionally, teacher ratings could provide unique information on child anxiety and/ or depressive symptoms that appear in a school context. Self-rated symptoms are important for identifying the subjective perspectives of children and are important to investigate, since a meta-analysis of parent-led CBT on child anxiety revealed significant effects according to parental ratings, but no corresponding effects based on anxiety outcomes reported by children [29]. As a future research direction, it would be beneficial to conduct larger-scale studies with increased sample sizes. Although we estimated the sample size apriori, the effect size was determined primarily for our primary outcome, which may have led to insufficient power to detect smaller differences between the intervention and control groups for all secondary outcomes. To achieve a more precise estimate, recent recommendations for pilot studies with continuous outcomes suggest that at least 70 participants should be included [56].

Modalities to increase parents’ engagement with the intervention should be investigated. Future research could compare the efficacy of Internet-delivered parent-led CBT with Internet-delivered CBT for children only. Additionally, further clinical trials could investigate the efficacy of this intervention in comparison to other treatments (e.g., traditional face-to-face therapy, psychoeducation) or as an add-on to traditional approaches. Finally, the cost-effectiveness of the program, as well as the mechanisms of change, needs to be investigated.

## Conclusion

ParentKIT is a promising intervention associated with improved child emotional problems as reported by parents and parental self-efficacy regarding parenting practices that can reduce risks associated with child anxiety and depression. This study is the first Internet-delivered intervention investigated with a Romanian sample of parents of children with elevated emotional problems. Further studies should investigate its long-term efficacy.

## Data Availability

The datasets analyzed during the current study are available from the corresponding author on reasonable request.

## References

[1] Polanczyk GV, Salum GA, Sugaya LS, Caye A, Rohde LA (2015). Annual Research Review: a meta-analysis of the worldwide prevalence of mental disorders in children and adolescents. J Child Psychol Psychiatry.

[2] Solmi M, Radua J, Olivola M, Croce E, Soardo L, Salazar de Pablo G (2022). Age at onset of mental disorders worldwide: large-scale meta-analysis of 192 epidemiological studies. Mol Psychiatry.

[3] Garber J, Weersing VR (2010). Comorbidity of anxiety and depression in Youth: implications for treatment and Prevention. Clin Psychology: Publication Div Clin Psychol Am Psychol Association.

[4] Cuijpers P, Karyotaki E, Eckshtain D, Ng MY, Corteselli KA, Noma H (2020). Psychotherapy for Depression Across different age groups: a systematic review and Meta-analysis. JAMA Psychiatry.

[5] Higa-McMillan CK, Francis SE, Rith-Najarian L, Chorpita BF (2016). Evidence base update: 50 years of research on treatment for child and adolescent anxiety. J Clin Child Adolesc Psychol.

[6] Rith-Najarian LR, Mesri B, Park AL, Sun M, Chavira DA, Chorpita BF (2019). Durability of cognitive behavioral Therapy effects for Youth and adolescents with anxiety, Depression, or traumatic stress:a Meta-analysis on Long-Term follow-ups. Behav Ther.

[7] Hansen AS, Telléus GK, Mohr-Jensen C, Lauritsen MB (2021). Parent-perceived barriers to accessing services for their child’s mental health problems. Child Adolesc Psychiatry Ment Health.

[8] Radez J, Reardon T, Creswell C, Lawrence PJ, Evdoka-Burton G, Waite P (2021). Why do children and adolescents (not) seek and access professional help for their mental health problems? A systematic review of quantitative and qualitative studies. Eur Child Adolesc Psychiatry.

[9] Stein K, Fazel M (2015). Depression in young people often goes undetected. Practitioner.

[10] Naslund JA, Aschbrenner KA, Araya R, Marsch LA, Unützer J, Patel V (2017). Digital technology for treating and preventing mental disorders in low-income and middle-income countries: a narrative review of the literature. Lancet Psychiatry.

[11] Patel V, Maj M, Flisher AJ, De Silva MJ, Koschorke M, Prince M (2010). Reducing the treatment gap for mental disorders: a WPA survey. World Psychiatry.

[12] Pipe A, Ravindran N, Paric A, Patterson B, Van Ameringen M, Ravindran AV (2022). Treatments for child and adolescent attention deficit hyperactivity disorder in low and middle-income countries: a narrative review. Asian J Psychiatry.

[13] Păsărelu CR, Andersson G, Bergman Nordgren L, Dobrean A (2017). Internet-delivered transdiagnostic and tailored cognitive behavioral therapy for anxiety and depression: a systematic review and meta-analysis of randomized controlled trials. Cogn Behav Ther.

[14] Eilert N, Wogan R, Leen A, Richards D (2022). Internet-delivered interventions for depression and anxiety symptoms in children and Young people: systematic review and Meta-analysis. JMIR Pediatr Parent.

[15] Cuijpers P, Karyotaki E, Ciharova M, Miguel C, Noma H, Stikkelbroek Y (2023). The effects of psychological treatments of depression in children and adolescents on response, reliable change, and deterioration: a systematic review and meta-analysis. Eur Child Adolesc Psychiatry.

[16] Warwick H, Reardon T, Cooper P, Murayama K, Reynolds S, Wilson C (2017). Complete recovery from anxiety disorders following cognitive behavior therapy in children and adolescents: a meta-analysis. Clin Psychol Rev.

[17] Tao Y, Zou X, Tang Q, Hou W, Wang S, Ma Z (2024). Mapping network connection and direction between anxiety and depression symptoms across the early, middle, and late adolescents: insights from a large Chinese sample. J Psychiatr Res.

[18] García-Escalera J, Chorot P, Valiente RM, Reales JM, Sandín B. Efficacy of transdiagnostic cognitive­-behavioral therapy for anxiety and depression in adults, children and adolescents: a meta­-analysis. Revista De Psicopatología Y Psicología Clínica. 2016;21:147–75.

[19] Carlucci L, Saggino A, Balsamo M (2021). On the efficacy of the unified protocol for transdiagnostic treatment of emotional disorders: a systematic review and meta-analysis. Clin Psychol Rev.

[20] Ewing DL, Monsen JJ, Thompson EJ, Cartwright-Hatton S, Field A (2015). A Meta-analysis of transdiagnostic cognitive behavioural therapy in the treatment of child and young person anxiety disorders. Behav Cogn Psychother.

[21] Păsărelu C-R, Dobrean A, Andersson G, Zaharie GC (2021). Feasibility and clinical utility of a transdiagnostic internet-delivered rational emotive and behavioral intervention for adolescents with anxiety and depressive disorders. Internet Interventions.

[22] Sandín B, García-Escalera J, Valiente RM, Espinosa V, Chorot P (2020). Clinical utility of an internet-delivered version of the Unified Protocol for Transdiagnostic Treatment of Emotional disorders in adolescents (iUP-A): a Pilot Open Trial. Int J Environ Res Public Health.

[23] Orgilés M, Morales A, Fernández-Martínez I, Méndez X, Espada JP (2023). Effectiveness of a transdiagnostic computerized self-applied program targeting children with emotional problems: a randomized controlled trial. J Affect Disord.

[24] Păsărelu C-R, Dobrean A, Păsărelu C-R, Dobrean A. Parental Involvement in Remotely Delivered CBT Interventions for Anxiety Problems in Children and Adolescents: A Systematic Review. New Developments in Anxiety Disorders. IntechOpen; 2016 [cited 2023 Jan 31]. Available from: https://www.intechopen.com/state.item.id.

[25] Lebowitz ER, Marin C, Martino A, Shimshoni Y, Silverman WK. Parent-based treatment as efficacious as cognitive behavioral therapy for childhood anxiety: a Randomized Noninferiority Study of supportive parenting for anxious childhood emotions. J Am Acad Child Adolesc Psychiatry. 2019;S0890-8567(19)30173-X. 10.1016/j.jaac.2019.02.014 PMC6732048 30851397

[26] Lebowitz ER, Omer H, Hermes H, Scahill L (2014). Parent training for childhood anxiety disorders: the SPACE Program. Cogn Behav Pract.

[27] Byrne G, Connon G, Martin E, McHugh S, Power L (2021). Evaluation of a parent-led cognitive behaviour therapy programme in routine clinical practice: a controlled trial. Br J Clin Psychol.

[28] Guzick AG, Leong AW, Dickinson EM, Schneider SC, Zopatti K, Manis J (2022). Brief, parent-led, transdiagnostic cognitive-behavioral teletherapy for youth with emotional problems related to the COVID-19 pandemic. J Affect Disord.

[29] Jewell C, Wittkowski A, Pratt D (2022). The impact of parent-only interventions on child anxiety: a systematic review and meta-analysis. J Affect Disord.

[30] Green I, Reardon T, Button R, Williamson V, Halliday G, Hill C (2023). Increasing access to evidence-based treatment for child anxiety problems: online parent-led CBT for children identified via schools. Child Adolesc Mental Health.

[31] Leonard RC, Guastello A, Cooke D, Storch EA, Lebowitz ER, Riemann BC (2021). An initial study of Symptom accommodation in adults with Depression. J Cogn Ther.

[32] McGorry PD, Mei C, Chanen A, Hodges C, Alvarez-Jimenez M, Killackey E (2022). Designing and scaling up integrated youth mental health care. World Psychiatry.

[33] Cobham VE, Radtke SR, Hawkins I, Jordan M, Ali NR, Ollendick TH (2024). Piloting a one-day parent-only intervention in the treatment of youth with anxiety disorders: child and family-level outcomes. Child Adolesc Psychiatry Mental Health.

[34] Faul F, Erdfelder E, Buchner A, Lang A-G (2009). Statistical power analyses using G*Power 3.1: tests for correlation and regression analyses. Behav Res Methods.

[35] Vlaescu G, Alasjö A, Miloff A, Carlbring P, Andersson G (2016). Features and functionality of the Iterapi platform for internet-based psychological treatment. Internet Interv.

[36] Goodman R (1997). The strengths and difficulties Questionnaire: A Research note. J Child Psychol Psychiatry.

[37] Stone LL, Otten R, Engels RCME, Vermulst AA, Janssens JMAM (2010). Psychometric properties of the parent and teacher versions of the strengths and difficulties Questionnaire for 4- to 12-Year-Olds: a review. Clin Child Fam Psychol Rev.

[38] Husky MM, Otten R, Boyd A, Pez O, Bitfoi A, Carta MG (2020). Psychometric properties of the strengths and difficulties Questionnaire in Children aged 5–12 years across seven European countries. Eur J Psychol Assess.

[39] Löwe B, Wahl I, Rose M, Spitzer C, Glaesmer H, Wingenfeld K (2010). A 4-item measure of depression and anxiety: validation and standardization of the Patient Health Questionnaire-4 (PHQ-4) in the general population. J Affect Disord.

[40] Caro-Fuentes S, Sanabria-Mazo JP. A Systematic Review of the Psychometric Properties of the Patient Health Questionnaire-4 in Clinical and Nonclinical Populations. Journal of the Academy of Consultation-Liaison Psychiatry. 2023 [cited 2024 Jan 31]; Available from: https://www.sciencedirect.com/science/article/pii/S2667296023008467. 10.1016/j.jaclp.2023.11.685 38012988

[41] Nicolas CC, Jorm AF, Cardamone-Breen MC, Lawrence KA, Yap MBH (2020). Parental self-efficacy for reducing the risk of adolescent depression and anxiety: Scale Development and Validation. J Res Adolescence.

[42] Larsen DL, Attkisson CC, Hargreaves WA, Nguyen TD (1979). Assessment of client/patient satisfaction: development of a general scale. Eval Program Plan.

[43] Andersson G, Käll A, Juhlin S, Wahlström C, de Fine Licht E, Färdeman S (2023). Free choice of treatment content, support on demand and supervision in internet-delivered CBT for adults with depression: a randomized factorial design trial. Behav Res Ther.

[44] Ellis A (1994). Reason and emotion in psychotherapy: revised and updated.

[45] Dryden W. The Distinctive Features of Rational Emotive Behavior Therapy. In: Bernard ME, Dryden W, editors. Advances in REBT: Theory, Practice, Research, Measurement, Prevention and Promotion. Cham: Springer International Publishing; 2019 [cited 2023 Jan 27]. p. 23–46. 10.1007/978-3-319-93118-0_2.

[46] Young P, Turner MJ (2023). To (i)B or not to i(B), that is the question: on the differences between Ellis’ REBT and Beck’s CT. Cogn Behav Therapist.

[47] Vîslă A, Flückiger C, Grosse Holtforth M, David D (2016). Irrational beliefs and psychological distress: a Meta-analysis. Psychother Psychosom.

[48] Team Rs (2019). RStudio. Boston, MA: Integrated Development for R. RStudio. Inc Version.

[49] van Buuren S, Karin G-O. mice: Multivariate Imputation by Chained Equations in R. Journal of Statistical Software. 2011 [cited 2020 Apr 6];045. Available from: https://econpapers.repec.org/article/jssjstsof/v_3a045_3ai03.htm.

[50] Rubin DB. Multiple imputation for survey nonresponse. 1987.

[51] Grund S, Robitzsch A, Luedtke O. mitml: Tools for Multiple Imputation in Multilevel Modeling. 2023 [cited 2023 Apr 20]. Available from: https://CRAN.R-project.org/package=mitml.

[52] Cohen J (1992). Statistical power analysis. Curr Dir Psychol Sci.

[53] Păsărelu C-R, David D, Dobrean A, Noje A, Șipoș R, Predescu E (2023). ADHDCoach—a virtual clinic for parents of children with ADHD: development and usability study. DIGITAL HEALTH.

[54] Breider S, de Bildt A, Nauta MH, Hoekstra PJ, van den Hoofdakker BJ (2019). Self-directed or therapist-led parent training for children with attention deficit hyperactivity disorder? A randomized controlled non-inferiority pilot trial. Internet Interv.

[55] Khor SPH, Fulgoni CM, Lewis D, Melvin GA, Jorm AF, Lawrence K (2022). Short-term outcomes of the therapist-assisted online parenting strategies intervention for parents of adolescents treated for anxiety and/or depression: a single-arm double-baseline trial. Aust N Z J Psychiatry.

[56] Teare MD, Dimairo M, Shephard N, Hayman A, Whitehead A, Walters SJ (2014). Sample size requirements to estimate key design parameters from external pilot randomised controlled trials: a simulation study. Trials.

